# Supplementary material for: Plasma midkine concentrations in healthy children, children with increased and decreased adiposity, and children with short stature
Source: PLoS One. 2019 Oct 24;14(10):e0224103. doi: 10.1371/journal.pone.0224103 (PMC6812815; doi:10.1371/journal.pone.0224103)
Supplement: Attachment — Submitted filename: Response to reviewers .docx [file pone.0224103.s002.docx]

August 5, 2016

Kathleen E. Bethin, MD, PhD

Academic Editor

*PLOS ONE*

Re: PONE-D-19-15659

Title: **Plasma midkine concentrations in healthy children, children with increased and decreased adiposity, and children with short stature**

Dear Dr. Bethin,

We would like to thank the reviewer and editor for their careful evaluation of our manuscript. As requested, we are providing the point-by-point response to the reviewer and editor and the revised version of our manuscript with track changes. The reviewer and editor made a number of important comments, which we carefully addressed, as detailed below. With these revisions, we believe that the paper is strengthened and therefore hope that the reviewer and editor will find that it is now suitable for publication in *PLOS ONE*.

Sincerely,

Jeffrey Baron, MD

Chief, Section on Growth and Development

**Reviewer comments:**

**Journal requirements #1:** Please ensure that your manuscript meets PLOS ONE's style requirements, including those for file naming.

**Response:** We believe that our manuscript meets PLOS One’s style including the file

names. Please let us know if any adjustments are needed.

**Journal requirements #2:** In your Methods section, please ensure you have also stated whether

you obtained consent from parents or guardians of the minors included in the

study.

**Response:** The language of consent process was revised as below.

*Page 4:* *Written informed consent was obtained from all subjects and from parents or*

*legal guardians of minors. Assent was also obtained from minors as appropriate.*

**Journal requirements #3**: “Data not shown” in your manuscript does not meet our data sharing requirements. PLOS does not permit references to inaccessible data.

**Response:** The “data not shown” is not critical to the manuscript and therefore was

simply deleted.

*Page 8:* *~~Plasma was obtained from subjects regardless of fasting or time of day because~~*

*~~these factors did not affect plasma MDK concentrations based on our evaluation~~*

*~~(data not shown). Measurement of MDK in serum did not show good parallelism~~*

*~~(data not shown) and the concentrations of MDK were far lower than in plasma,~~*

*~~therefore only plasma MDK concentrations are reported.~~*

**Editor comments #1:** The authors state, “All relevant data are within the manuscript and its Supporting Information files.” However, the raw data are not included in the manuscript and there are no supporting files.

**Response:** The raw data in excel is now submitted as a supporting information file.

**Editor comments #2:** Fig 1 legend does not belong on the figure itself.

Response: The fig 1 legend is revised as below.

*Page 4:*
***Fig 1****.* ***Subject groups.*** *Healthy children without obesity, children and young adults with abnormal adiposity (obesity and anorexia nervosa), children with short stature, and healthy adults were studied. Numbers of subjects in each group are shown in parentheses. SS, short stature; AN, anorexia nervosa; ISS, idiopathic short stature; SGA, small for gestational age; GH, growth hormone; NIH, National Institutes of Health; MGH, Massachusetts General Hospital; U.S., United States (studied at NIH)*

**Editor comment #3:** Fig 2 legend- must be reworked. A figure legend is not a description of the results. Please refer to reviewer comments.

**Response:** Thank you for the editor’s and reviewer’s comment. Fig 2 legend is revised

as below.

*Page 10:*

***Fig 2. Plasma midkine (MDK) concentrations in healthy children.*** *Plasma was obtained from healthy, non-obese children younger than 18 years (n=222) from the United States or South Korea. Plasma MDK concentration was measured by sandwich enzyme-linked immunosorbent assay (ELISA). A) Scatterplot of plasma midkine (MDK) concentrations (ng/mL) vs age. B) MDK concentrations (mean ± SEM) stratified by age. C) Scatterplot of plasma MDK concentrations vs. height standard deviation score (SDS) and D) Scatterplot of plasma MDK concentrations vs. body mass index (BMI) SDS. E) Plasma MDK concentrations (mean ± sem) stratified by Tanner stage in those subjects who had pubertal staging performed at the time of blood collection (n = 132).*

**Editor comment #4:** For Fig 2 B please explain why/how the age brackets shown were chosen.

**Response:** The following sentence was added to the methods section:

***Page9****:*

*In healthy children, the relationship between plasma MDK concentration and age was analyzed both by treating age as a continuous variable and also by categorizing for age: infancy (< 1 year), prepubertal age range (1-7 years), peripubertal to pubertal age range (8-14 years), and late pubertal to postpubertal age range (15-18 years).*

**Editor comment #5:** I agree with reviewer #1 assessment- The results section does not flow with the numbered sections. I would remove the numbers and the titles and add some transitions from one section to the other.

**Response**: As suggested, the numbers and the titles in result section were removed and

the transitions were revised.

**Editor comment #6:** On page 11, lines 240-242 the MDK in children with ISS is compared to “healthy children.” This is implying that children with ISS are not healthy. I would suggest rewording the sentence.

**Response**: Thank you for the editor’s comment. We revised the sentence as below.

*Page 11: To explore the relationship between MDK and linear growth, plasma midkine concentrations were measured in 68 children with idiopathic short stature (ISS, median height SDS -2.2) and compared to healthy children with stature within the normal range.*

Lastly, we would like to inform the editor and reviewer that we revised Fig 1 for Y-axis to contain raw data in a log scale (not log-transformed data) to help readers.

**Reviewer #1:** The submitted manuscript by Lee et al describes plasma midkine levels in different cohorts of children with normal weight, obesity and underweight, as well as short stature. This study was done because of prior observation that midkine levels were higher in obesity and short stature. The authors set out to clarify the midkine concentrations in these populations. The cohorts are well characterized and described. The data is clear and the manuscript is well-written. There a few items which could be addressed by the authors.

1) Page 7, line 133. The authors describe the Tanner staging for the pediatric cohort. However, they used different Tanner staging procedures based on sex -- females were staged for gonadarche, while males were staged by pubarche. The authors then evaluated midkine levels based on Tanner stage. However, the authors are combining 2 different pubertal assessments into one category and gonadarche is biochemically different than pubarche. The authors should discuss the rationale for this categorization. Alternatively, if they have data that would be consistent across both sexes, then midkine levels could be presented based on that Tanner staging.

**Response**: We agree with the reviewer’s point. The data were obtained from subject’s medical record at the time of blood collection and breast development for girls and pubic hair for boys were primarily available. To address the reviewer’s point, we revised our manuscript as follows.

*Page 8:*

*For 131 healthy children without obesity, Tanner staging was assessed by physical exam conducted by an experienced pediatric endocrine research nurse or pediatric endocrinologist. The Tanner stages were later obtained from the subjects’ records. Breast development for girls and pubic hair for boys were used for analysis because these were the observations most widely recorded.*

2) Page 8, line 169. What does the term "parallelism" mean ? Do they mean to say correlation or consistency?

**Response:** We used the term “parallelism” to refer to a desirable property of an immunoassay. To assess parallelism, a subject’s plasma with high endogenous MDK is serially diluted and then assayed. If the measured concentrations of MDK decrease proportionally to the dilution factor, then the assay is said to be parallel, indicating that the binding characteristic of the antibodies to endogenous MDK is the same as antibody binding to the calibrator MDK and that the blood plasma does not create a matrix effect which alters the binding. However, in the revised manuscript, we decided not to show the parallelism data because it is not critical to the study, and we therefore deleted the sentence referring to parallelism.

3) Results (starting on page 9) Do the individual results categories need to be numbered??

**Response**: We agree that the numbering is not necessary, and therefore we removed the numbers and subtitles and instead used the first sentence of each paragraph to aid the reader with the transitions.

4) Figure legend for Fig 2 should be reworked. Would recommend as follows: This figure legend could be reworked

**Response**: We appreciate the reviewer’s comment and revised the legend as suggested.

***Fig 2. Plasma midkine concentrations in healthy children.*** *Plasma was obtained from healthy, non-obese children younger than 18 years (n=222) from the United States or South Korea. Plasma midkine concentration was measured by sandwich enzyme-linked immunosorbent assay (ELISA) A) Scatterplot of plasma midkine (MDK) concentrations (ng/mL) vs age. B) MDK concentrations (mean ± SEM) stratified by age. C) Scatterplot of plasma MDK concentrations vs. height standard deviation score (SDS) and D) Scatterplot of plasma MDK concentrations vs. body mass index (BMI) SDS. E) Plasma MDK concentrations (mean ± sem) stratified by Tanner stage* *in those subjects who had pubertal staging performed at the time of blood collection (n = 132).*
